# A novel Hemoglobin-Albumin-Lymphocyte-Platelet (HALP)-based prognostic model to predict the progression of premenopausal breast cancer: a retrospective cohort study

**DOI:** 10.1186/s12885-026-16642-7

**Published:** 2026-07-29

**Authors:** Sirui Zhang, Lingyun Xu, Wenting Peng

**Affiliations:** Department of Breast Surgery, Changzhou Medical Center, The Second People’s Hospital of Changzhou, The Third Affiliated Hospital of Nanjing Medical University, Nanjing Medical University, Changzhou, Jiangsu P.R. China

**Keywords:** HALP, Premenopausal breast cancer, Prognostic models, Immunohistochemical biomarker, Progression-free survival

## Abstract

**Background:**

The incidence of breast cancer is increasing annually in premenopausal patients, necessitating an improved prognostic prediction model. The hemoglobin-albumin-lymphocyte-platelet (HALP) serves as a comprehensive index that assesses systemic inflammatory response, nutritional status, and immune function.

**Methods:**

A total of 310 patients with invasive breast cancer (IBC) were enrolled, binary logistic regression and Cox multivariate analysis were applied to determine the risk factors for recurrence or metastasis, and the optimal cut-off value was determined using the Youden index. The prediction performance was compared using the Kaplan-Meier method and the area under the receiver operating characteristic curve (AUC).

**Results:**

In the premenopausal cohort, Cox multivariate analysis demonstrated that TNM stage (HR = 2.33, 95% CI: 1.264–4.28, *P* = 0.007), and HALP (HR = 0.25, 95%CI: 0.095–0.64, *P* = 0.004) were independent prognostic factors for progression-free survival (PFS). Positive epidermal growth factor receptor (EGFR) (*P* < 0.0001) and Cytokeratin 5/6 (CK5/6) expression (*P* = 0.0048), higher TNM stage (*P* < 0.0001), and lower HALP (*P* < 0.0001) were significantly associated with worse PFS. A prediction model for IBC was established based on HALP, EGFR, and CK5/6. The AUC of the combined HALP-EGFR model was 0.764, which was better than single predictive model, as well as the traditional TNM stage model. Moreover, a comprehensive model integrating TNM stage, EGFR, CK5/6, and HALP achieved an even higher AUC of 0.844.

**Conclusion:**

Our results indicate that the HALP-based prognostic model, particularly when combined with EGFR and further integrated with TNM stage and CK5/6, demonstrates high predictive value in premenopausal IBC patients, providing valuable guidance for individualized treatment.

**Ethics approval and registration:**

The study was reviewed and approved by the Ethics Committee of The Affiliated Changzhou No. 2 People’s Hospital of Nanjing Medical University (2018KY039_01). Date: January 1st, 2018.

**Supplementary Information:**

The online version contains supplementary material available at https://doi.org/10.1186/s12885-026-16642-7.

## Background

Breast cancer is the most common malignancy in women [1], and on top of that, the incidence of breast cancer in the premenopausal population has increased significantly in recent years [2]. Premenopausal breast cancer not only tends to be more aggressive than postmenopausal breast cancer, it features distinct pathogenesis, risk factors, and clinical management. Therefore, developing a convenient and precise prognostic model for premenopausal women with breast cancer to optimize individual treatment is crucial.

The immunosuppressive state in premenopausal breast cancer can lead to tumor immune evasion, thereby promoting tumor progression. It can occur due to chronic inflammation and malnutrition, increasing the risk of infection and death in patients with chronic diseases [3]. Numerous studies have indicated that neutrophils, platelets, monocytes, and other inflammatory cells play important roles in tumor progression and metastasis [4–6]. Our previous research also showed the effect of chronic inflammation in the development and progression of breast cancer [7]. The role of preoperative peripheral blood biomarkers, such as the neutrophil-to-lymphocyte ratio (NLR), platelet-to-lymphocyte ratio (PLR), monocyte-to-lymphocyte ratio (MLR), systemic immune-inflammation index (SII), and prognostic nutritional index (PNI), have been used to explore the relationship between physiological and disease status and to predict PFS in patients with cancer, including colorectal and lung cancer [8].

HALP, an emerging prognostic indicator developed in recent years [9], integrates systemic inflammation, nutritional status, and immune function in tumor patients. This composite biomarker reflects tumor tolerance based on preoperative peripheral blood parameters. EGFR and CK5/6, which serve as key molecular biomarkers in breast cancer, also show significant implications in the diagnosis and prognosis of breast cancer. Previous studies have confirmed the significant correlation of EGFR and CK5/6 expression and progression of breast cancer [10–14]. However, the predictive efficacy of these biomarkers for breast cancer requires further improvement.

Since standalone prognostic markers exhibited inherent limitations, our study intended to construct a combined prognostic model in breast cancer patients. In this study, we systematically analyzed the independent and combined prognostic efficacy of these biomarkers.

## Materials and methods

### Patient samples and study design

**Fig. 1:**
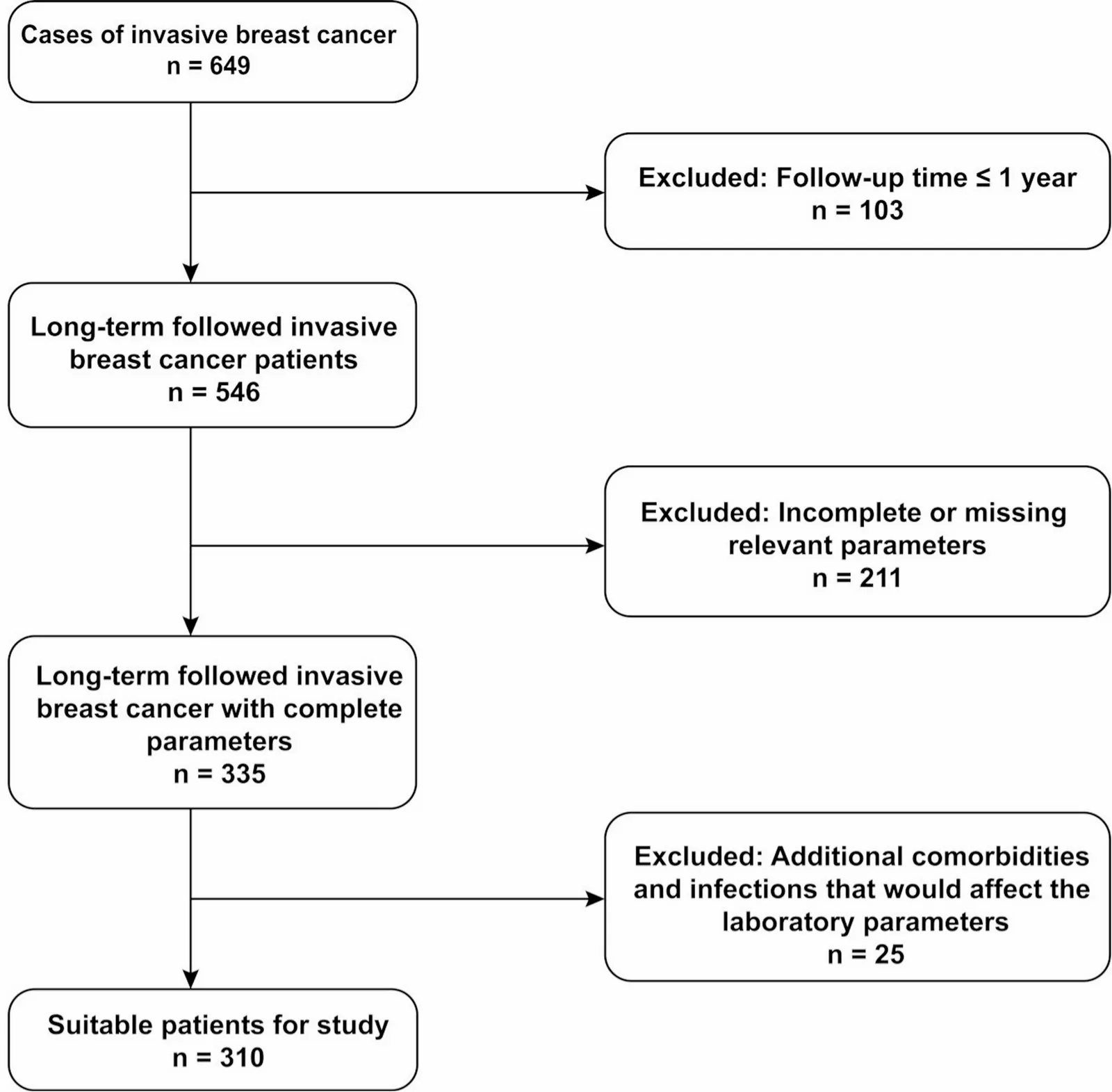
Flow chart for screening patients

From January 2015 to December 2020, 649 patients with invasive breast cancer who underwent surgery at Changzhou Second People’s Hospital were screened based on the flowchart (Fig. 1). Patients were excluded on the following conditions: (1) a follow-up time less than one year, except for patients with recurrence or metastasis; (2) incomplete or missing clinical parameters; (3) major organ failure; (4) severe infections that affected laboratory parameters; (5) those who received preoperative anti-tumor therapy (including radiation, chemotherapy, targeted therapy, immunotherapy, interventional therapy, and traditional Chinese medicine). The date of recurrence or metastasis was defined as the date when recurrence or metastasis was confirmed by biopsy or clinically. PFS was defined as the interval from diagnosis to the first documented recurrence, metastasis, or death from any cause. For patients with non-metastatic disease at diagnosis (stage I–III), recurrence or metastasis was defined as the first evidence of local recurrence, regional lymph node recurrence, or distant metastasis confirmed by imaging or biopsy. For the two patients with de novo stage IV disease at diagnosis, PFS was defined as the time from diagnosis to the first disease progression or death. Patients without events were censored at the time of last follow-up.

### Data collection and follow-up

Clinical data were collected from patients, including clinicopathological parameters, such as age, body mass index (BMI), TNM stage, molecular subtype, immunohistochemical markers, and preoperative routine blood examination results.

Preoperative peripheral blood samples were uniformly collected within 7 days before surgery, prior to any anti-tumor treatment. The BMI, NLR, PLR, MLR, SII, HALP, and PNI were calculated using the following formulae: BMI = weight (kg) / height^2^ (m); NLR = neutrophil count (n/mm^3^) / lymphocyte count (n/mm^3^); PLR = platelet count (n/mm^3^) / lymphocyte count (n/mm^3^); MLR = monocyte count (n/mm^3^) / lymphocyte count (n/mm^3^); SII = platelet count (n/mm^3^) × neutrophil count (n/mm^3^) / lymphocyte count (n/mm^3^); HALP = hemoglobin (g/L) × albumin (g/L) × lymphocytes (/L) / platelets (/L) and PNI = albumin level (g/L) + 5 × lymphocyte count (n/mm^3^).

Patients should undergo a comprehensive physical examination according to clinical guidelines. Standard follow-up includes breast and lymph node ultrasonography, mammography, bone mineral density, computed tomography, magnetic resonance imaging (including bones, head, and liver), routine blood examination, and tumor markers.

### Statistical analysis

Statistical analyses were performed using SPSS 26.0 (SPSS, Inc., Chicago, IL) and R software (http://www.R-project.org; version 4.4.0). We used binary logistic regression analysis to analyze the impact of relevant factors on postoperative recurrence and metastasis in IBC patients. According to the receiver operating characteristic (ROC) curve, the optimal cut-off value was determined using the Youden index. Cox regression analysis was used to determine the risk factors. Prediction performance was compared using the Kaplan-Meier method and AUC. A value of *P* < 0.05 was considered statistically significant. To assess internal validity and avoid overfitting, we performed bootstrap internal validation with 500 resamples. For each of the three candidate prognostic models (HALP-EGFR, HALP-CK5/6, EGFR-CK5/6), we repeatedly (500 times) drew bootstrap samples (with replacement) from the cohort, refitted the Cox proportional hazards model, and evaluated its discriminative ability by calculating the AUC for 3-year PFS using the original dataset as the validation set. The mean AUC, standard deviation (SD), and bias-corrected 95% confidence intervals (percentile method) were reported. A mean AUC > 0.7 was considered acceptable discrimination, and > 0.8 as good.

## Results

### Patient characteristics

The clinicopathological characteristics of the 310 patients with IBC are presented in Table 1. The average age of the study population was 57.5 (range: 27–98) years, and the median follow-up period was 68 (range: 1-118) months. Overall, 37 patients experienced recurrence or metastasis, 48.4% (150/310) were premenopausal, only 15.4% (48/310) of patients were at a higher TNM stage (stage III and stage IV). Postoperative immunohistochemistry (IHC) showed that CK5/6 expression was negative in 82.6% (256/310) of patients, and EGFR expression was negative in 61.9% (192/310). The mean ± SD values of HALP, PNI, PLR, MLR, NLR, and SII are shown in Table 1.

**Table 1:** The Clinicopathological characteristics of patients with IBC (*n* = 310)

Characteristics	*N* = 310
Age, years	
< 45	83 (26.8%)
≥ 45	227 (73.2%)
BMI	
< 24	137 (44.2%)
≥ 24	173 (55.8%)
Menopause status	
premenopausal	150 (48.4%)
postmenopausal	160 (51.6%)
TNM stage	
I	113 (36.5%)
II	149 (48.1%)
III	46 (14.8%)
IV	2 (0.6%)
Molecular typing	
Luminal	235 (75.8%)
HER2	46 (14.8%)
TNBC	29 (9.4%)
Lymph node status	
N^−^	168 (54.2%)
N^+^	142 (45.8%)
HR	
negative	75 (24.2%)
positive	235 (75.8%)
HER2	
negative	125 (40.3%)
positive	185 (59.7%)
Ki-67	
< 14	62 (20.0%)
≥ 14	248 (80.0%)
Recurrence or metastasis	
yes	37 (11.9%)
no	273 (88.1%)
CK5/6	
negative	256 (82.6%)
positive	54 (17.4%)
EGFR	
negative	192 (61.9%)
positive	118 (38.1%)
HALP	43.63 ± 24.05
PNI	50.21 ± 4.48
PLR	138.14 ± 56.80
MLR	0.24 ± 0.14
NLR	3.06 ± 3.05
SII	661.93 ± 619.01

*BMI* body mass index, *CK5/6* Cytokeratin 5/6, *EGFR* epidermal growth factor receptor, *HALP* hemoglobin, albumin, lymphocyte, and platelet, *IBC* invasive breast cancer, *MLR* monocyte-to-lymphocyte ratio, *NLR* neutrophil-to-lymphocyte ratio, *PLR* platelet-to-lymphocyte ratio, *PNI* prognostic nutritional index, *SII* systemic immune-inflammation index

### Analysis of the impact of relevant factors on prognosis in different cohorts

The population was stratified based on age, BMI, presence of lymph node metastasis, Ki-67 expression, menopausal status, hormone receptor status, and molecular subtypes. Binary logistic regression analysis was performed to analyze the relationship between the above indicators and postoperative recurrence or metastasis in different populations with IBC. The main findings are listed in Table 2, with extended results available in Table S1. We found EGFR (*P* < 0.0001), CK5/6 (*P* = 0.008), HALP (*P* = 0.035), and PLR (*P* = 0.043) were significantly associated with postoperative recurrence or metastasis in premenopausal women.

**Table 2:** Binary logistic regression analysis for PFS in premenopausal and postmenopausal cohort

Cohort	Indicators	OR (95% CI)	*P* value
Premenopausal	EGFR	5.924 (2.225–15.772)	< 0.0001
	CK5/6	3.738 (1.411–9.908)	0.008
	HALP	0.962 (0.928–0.997)	0.035
	PNI	0.953 (0.863–1.053)	0.348
	MLR	0.977 (0.060-15.838)	0.987
	PLR	1.010 (1.000-1.020)	0.043
	NLR	1.002 (0.875–1.147)	0.981
	SII	1.000 (0.999–1.001)	0.716
Postmenopausal	EGFR	2.125 (0.718–6.286)	0.173
	CK5/6	1.398 (0.365–5.360)	0.625
	HALP	1.013 (0.997–1.029)	0.102
	PNI	0.967 (0.853–1.097)	0.606
	MLR	1.354 (0.017-109.272)	0.893
	PLR	0.991 (0.978–1.004)	0.161
	NLR	0.835 (0.601–1.159)	0.281
	SII	0.999 (0.998–1.001)	0.277

*CI* confidence interval, *CK5/6* Cytokeratin 5/6, *EGFR* epidermal growth factor receptor, *HALP* hemoglobin, albumin, lymphocyte, and platelet, *MLR* monocyte-to-lymphocyte ratio, *NLR* neutrophil-to-lymphocyte ratio, *OR* odds ratio, *PFS* progression-free survival, *PNI* prognostic nutritional index, *SII* systemic immune-inflammation index

### Premenopausal cohort characteristics

**Fig. 2:**
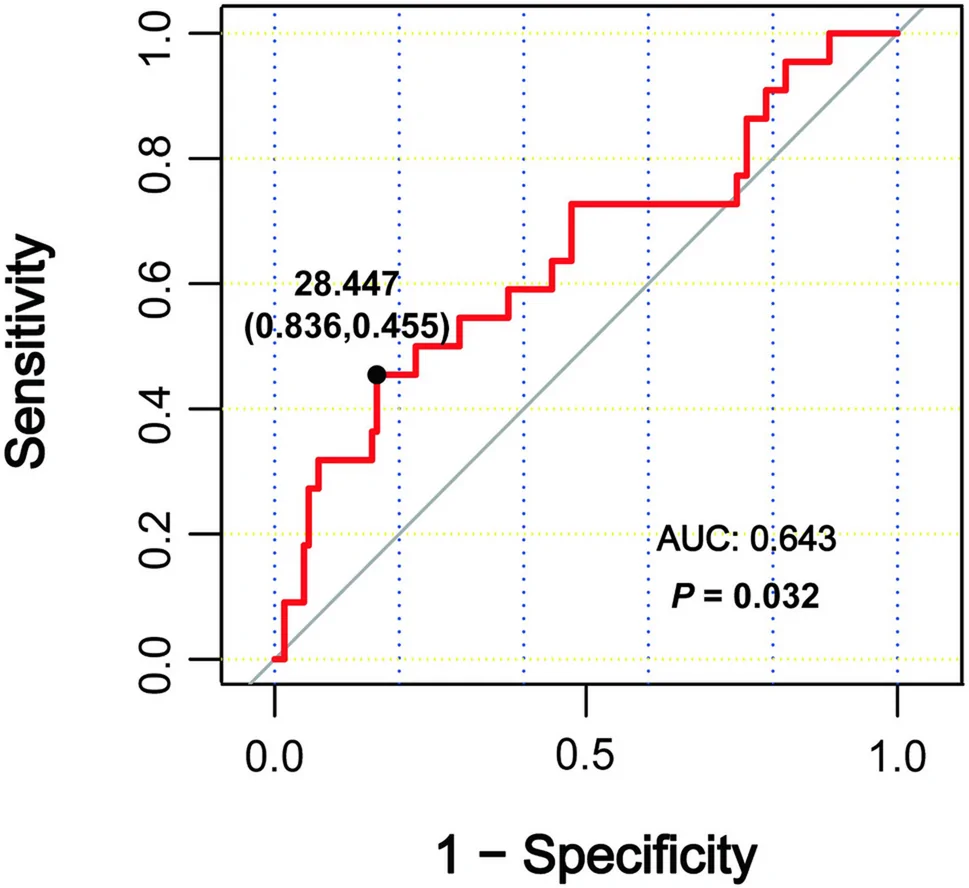
ROC curves and AUC of HALP score for predicting PFS. AUC, area under the curve; HALP, hemoglobin, albumin, lymphocyte, and platelet; PFS, progression-free-survival; ROC, receiver operating characteristic

Based on the above findings, we focused on the premenopausal cohort to develop a novel prognostic model. The clinicopathological characteristics of the 150 premenopausal IBC patients in the premenopausal cohort are listed in Table 3. The median age of the study population was 41.0 years (range: 27–58), and the median follow-up period was 74 months (range: 3–114). Among them, 22 patients experienced recurrence or metastasis. Postoperative immunohistochemistry revealed that CK5/6 expression was negative in 80.7% (121/150) of patients, and EGFR expression was negative in 67.3% (101/150). The mean ± SD values of HALP and PLR were 40.60 ± 15.20 and 140.92 ± 46.23, respectively. ROC curve analysis demonstrated that HALP had a sensitivity of 0.455, specificity of 0.836, Youden’s index of 0.291, an AUC of 0.643, and the optimal cut-off value for HALP was determined to be 28.447 (Fig. 2). However, the ROC curve analysis for PLR showed no statistical significance.

**Table 3:** The Clinicopathological characteristics of premenopausal patients with IBC (*n* = 150)

Premenopausal cohort characteristics	*N* = 150
Age, years	
< 45	81 (54.0%)
≥ 45	69 (46.0%)
BMI	
< 24	53 (35.3%)
≥ 24	97 (64.7%)
TNM stage	
I	44 (29.3%)
II	81 (54.0%)
III	24 (16.0%)
IV	1 (0.7%)
Molecular typing	
Luminal	116 (77.3%)
HER2	22 (14.7%)
TNBC	12 (8.0%)
Lymph node status	
N^−^	77 (51.3%)
N^+^	73 (48.7%)
HR	
negative	34 (22.7%)
positive	116 (77.3%)
HER2	
negative	59 (39.3%)
positive	91 (60.7%)
Ki-67	
< 14	27 (18.0%)
≥ 14	123 (82.0%)
Recurrence or metastasis	
yes	22 (14.7%)
no	128 (85.3%)
CK5/6	
negative	121 (80.7%)
positive	29 (19.3%)
EGFR	
negative	101 (67.3%)
positive	49 (32.7%)
HALP	40.60 ± 15.20
PLR	140.92 ± 46.23

*CK5/6* Cytokeratin 5/6, *EGFR* epidermal growth factor receptor, *HALP* hemoglobin, albumin, lymphocyte, and platelet, *IBC* invasive breast cancer, *PLR* platelet-to-lymphocyte ratio

### Determination and validation of the risk factors

**Fig. 3:**
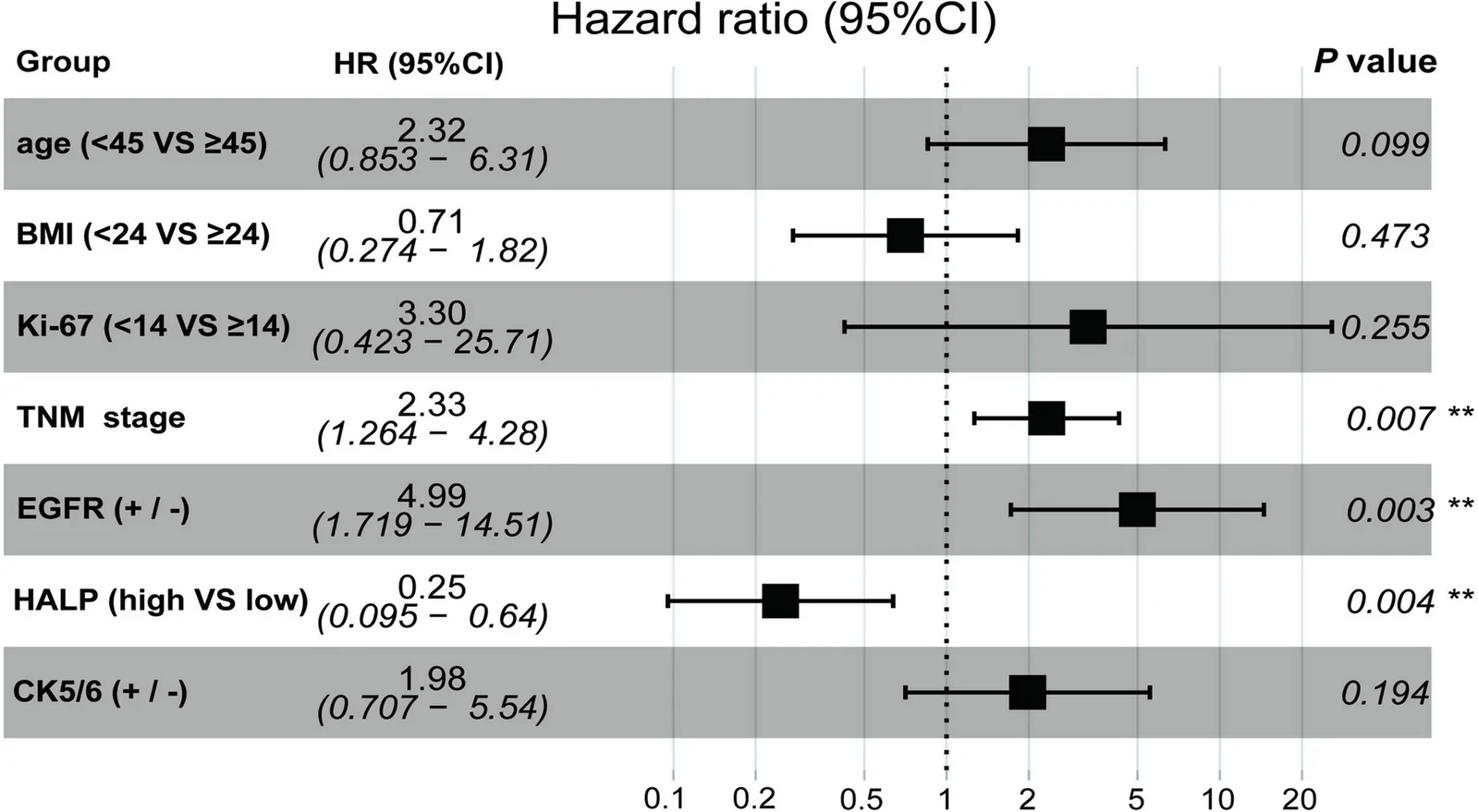
Cox multivariate analysis of factors associated with survival time for patients with IBC in the premenopausal cohort. CI, confidence interval; CK5/6, Cytokeratin 5/6; EGFR, epidermal growth factor receptor; HALP, hemoglobin, albumin, lymphocyte, and platelet; HR, hazard ratio; IBC, invasive breast cancer

**Fig. 4:**
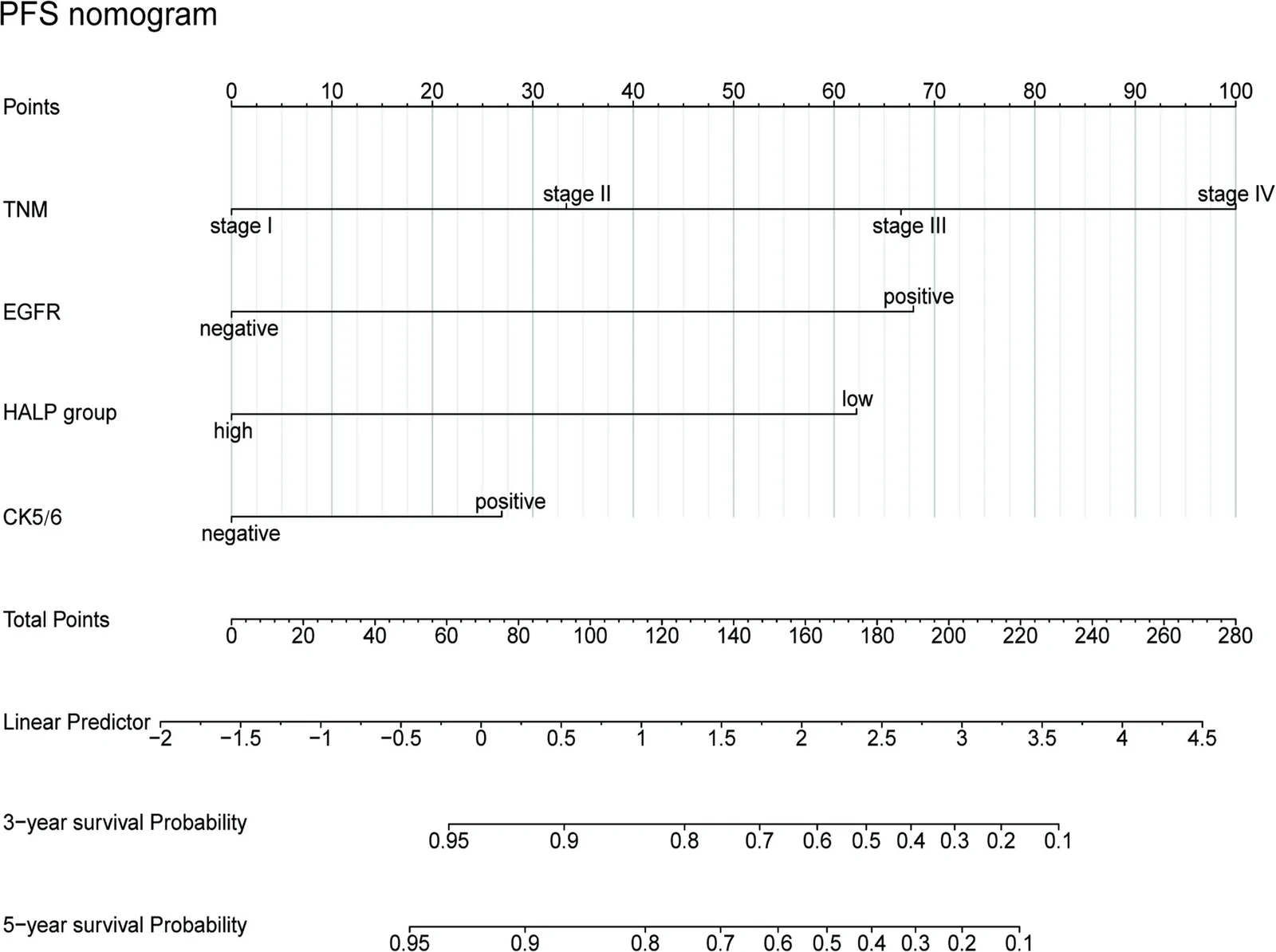
The Nomogram for predicting the 3-year and 5-year PFS of IBC in the premenopausal cohort. CK5/6, Cytokeratin 5/6; EGFR, epidermal growth factor receptor; HALP, hemoglobin, albumin, lymphocyte, and platelet; IBC, invasive breast cancer; PFS, progression-free survival

**Fig. 5:**
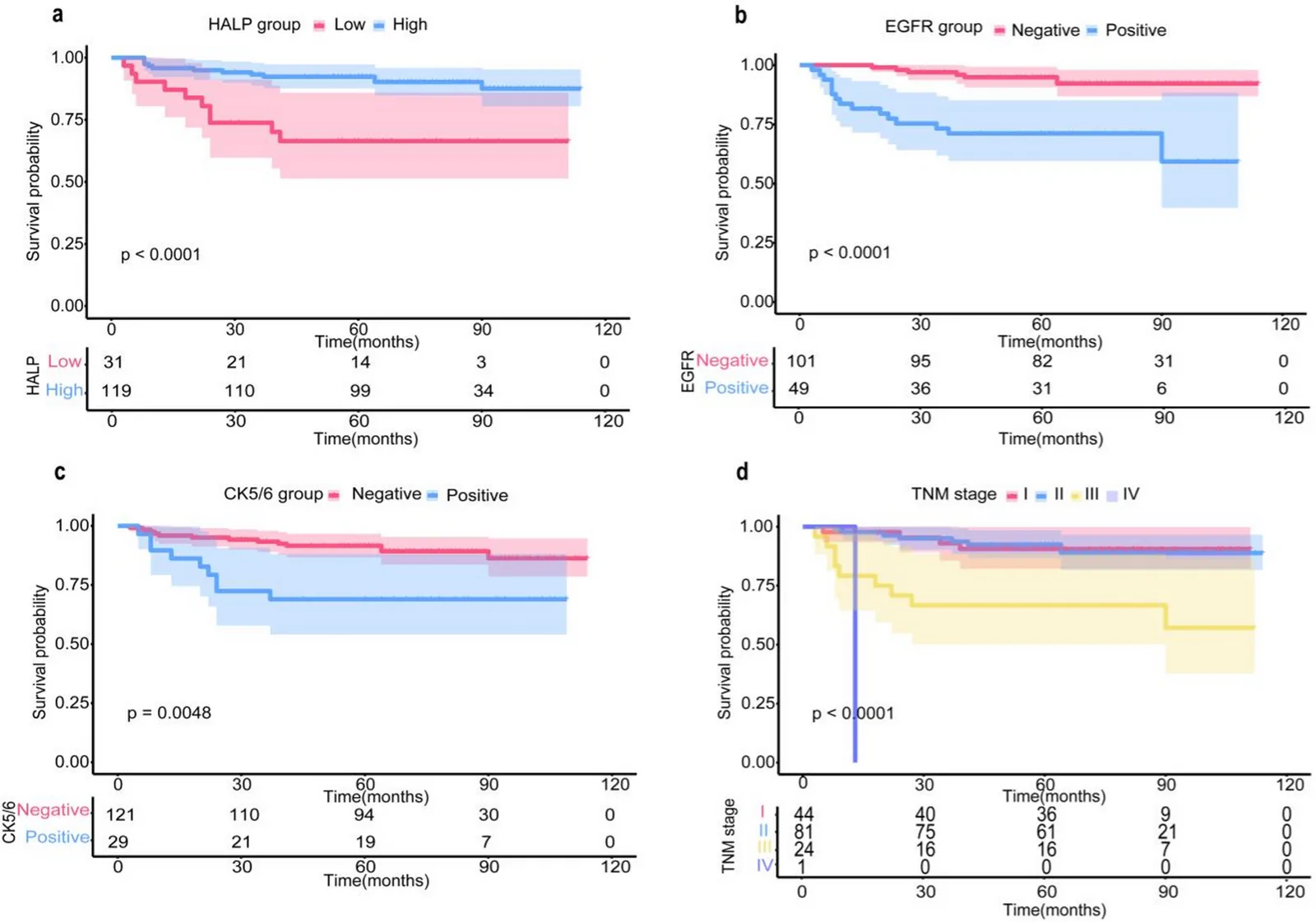
Kaplan-Meier curves for cancer survival in patients with IBC in the premenopausal cohort. **a** Kaplan–Meier plots of the combined model illustrating that the patients in the low HALP group showed poorer PFS than those in the high HALP group. **b** Kaplan–Meier plots of the single model illustrating that the patients in the positive EGFR group showed poorer PFS than those in the negative EGFR group. **c** Kaplan–Meier plots of the single model illustrating that the patients in the positive CK5/6 group showed poorer PFS than those in the negative CK5/6 group. **d** Kaplan–Meier plots of the single model illustrating that the patients in the high TNM stage group showed poorer PFS than those in the low TNM stage group. CK5/6, Cytokeratin 5/6; EGFR, epidermal growth factor receptor; HALP, hemoglobin, albumin, lymphocyte, and platelet; IBC, invasive breast cancer; PFS, progression-free survival

We stratified the patients into high and low HALP groups according to the cut-off value. Multivariate Cox analysis demonstrated that TNM stage (HR = 2.33, 95% CI: 1.264–4.28, *P* = 0.007), epidermal growth factor receptor (HR = 4.99, 95% CI: 1.719–14.51], *P* = 0.003), and HALP (HR = 0.25, 95% CI: 0.095–0.64, *P* = 0.004) were independent prognostic factors for PFS in the premenopausal cohort with IBC (Fig. 3). However, after additional adjustment for molecular subtype, EGFR was no longer statistically significant (HR = 4.99, 95% CI: 1.72–14.51, *P* = 0.076), while age showed a strong association with PFS. The adjusted results are presented in Table S2. Although CK5/6 and EGFR were not independent prognostic factors for PFS, binary logistic regression analysis demonstrated a significant association with postoperative recurrence or metastasis in patients with IBC. Based on the multivariate Cox regression analyses, nomograms for the prediction of the 3- and 5-year incidence were constructed based on the above prognostic factors, including TNM stage, EGFR, HALP, and CK5/6 (Fig. 4). The subsequent Kaplan-Meier method was used to analyze survival time. The results demonstrated that positive EGFR (*P* < 0.0001) and Cytokeratin 5/6 expression (*P* = 0.0048), higher TNM stage (*P* < 0.0001), and lower HALP (*P* < 0.0001) were significantly associated with worse PFS (Fig. 5).

### Establishment and evaluation of predictive models

**Fig. 6:**
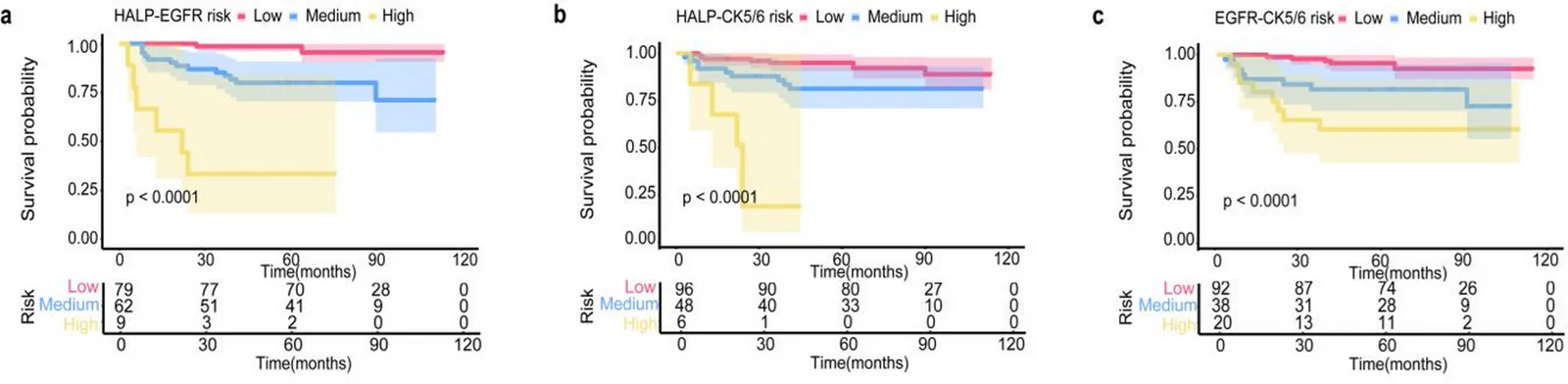
Kaplan-Meier curves for cancer survival in patients with IBC in the premenopausal cohort. **a** Kaplan–Meier plots of the combined model illustrating that the patients in the high-risk group showed poorer PFS than those in the low-risk group in HALP-EGFR model. **b** Kaplan–Meier plots of the combined model illustrating that the patients in the high-risk group showed poorer PFS than those in the low-risk group in EGFR-CK5/6 model. **c** Kaplan–Meier plots of the combined model illustrating that the patients in the high-risk group showed poorer PFS than those in the low-risk group in HALP-CK5/6 model. CK5/6, Cytokeratin 5/6; EGFR, epidermal growth factor receptor; HALP, hemoglobin, albumin, lymphocyte, and platelet; IBC, invasive breast cancer; PFS, progression-free survival

**Fig. 7:**
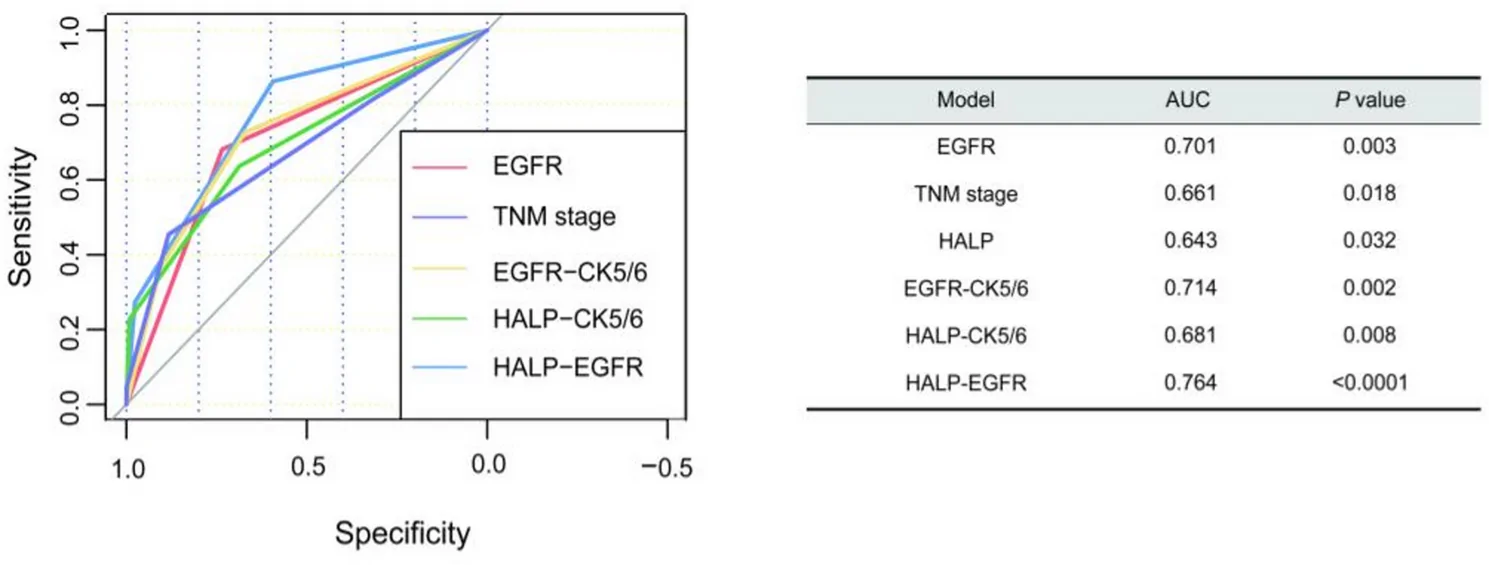
Comparison of single and multiple models in predicting progression. The AUC of EGFR, TNM stage, HALP, HALP-EGFR, EGFR-CK5/6, and HALP-CK5/6 models. AUC, area under the curve; CK5/6, Cytokeratin 5/6; EGFR, epidermal growth factor receptor; HALP, hemoglobin, albumin, lymphocyte, and platelet

Premenopausal patients with IBC were stratified by HALP and IHC biomarkers to assess the risk of postoperative recurrence or metastasis (Table 4). Kaplan-Meier method showed that high-risk groups in the HALP–EGFR (*P* < 0.0001), HALP–CK5/6 (*P* < 0.0001), and EGFR–CK5/6 (*P* < 0.0001) models had significantly poorer PFS (Fig. 6). Although the high-risk group showed significantly poorer PFS, the sample size of this subgroup was small, which limits the statistical robustness of the comparison. Therefore, these results should be interpreted cautiously and considered hypothesis-generating rather than definitive. Meanwhile, due to the small sample size in some risk subgroups, we combined the ‘medium’- and ‘high-risk’ groups into a single ‘non-low-risk’ group to ensure statistical robustness. The corresponding KM curves are provided in the Supplementary Materials (Figures S1). Meanwhile, ROC curves were used to evaluate the predictive performance of each model. The AUCs of the EGFR, HALP-EGFR, HALP-CK5/6, EGFR-CK5/6, HALP, and TNM stage models were 0.701, 0.764, 0.681, 0.714, 0.661 and 0.643, respectively (Fig. 7). The internal validation results of the three prognostic models are summarized as follows: the HALP-EGFR model demonstrated good discriminative ability with a mean bootstrap-corrected 3-year AUC of 0.831 (95% CI: 0.810–0.844, SD = 0.018). The EGFR-CK5/6 model showed moderate to good discrimination (mean AUC = 0.802, 95% CI: 0.770–0.811, SD = 0.014). The HALP-CK5/6 model yielded a mean AUC of 0.727 (95% CI: 0.728–0.731, SD = 0.022). The narrow confidence intervals and small standard deviations across all models indicate high stability and low sampling variability, suggesting that the observed performance is not driven by a particular subset of patients. These findings support that the models are not severely overfitted and maintain robust predictive capacity in internal validation. To further improve prognostic performance, we constructed a comprehensive prediction model by incorporating all variables with significance or a trend in multivariate analysis (TNM stage, EGFR, CK5/6, and HALP). The predicted probability was calculated, and the ROC curve was plotted (Figure S2). The comprehensive model achieved an AUC of 0.844 (95% confidence interval: 0.743–0.932, based on 2000 stratified bootstrap replicates), which was higher than that of single models and all two-parameter combinations. These results indicate that integrating anatomic stage, molecular biomarkers, and systemic inflammatory-nutritional indices provides the best prognostic discrimination for premenopausal IBC patients.

**Table 4:** Multiple models based on the HALP and IHC biomarkers

Prognostic Model	Risk Group	Factor1	Factor2
EGFR-CK5/6	Low	Negative EGFR	Negative CK5/6
	Medium	Negative for any index	
	High	Positive EGFR	Positive CK5/6
HALP-EGFR	Low	High HALP (HALP > 28.447)	Negative EGFR
	Medium	High HALP and positive EGFR or low HALP and negative EGFR	
	High	Low HALP (HALP < = 28.447)	Positive EGFR
HALP-CK5/6	Low	High HALP (HALP > 28.447)	Negative CK5/6
	Medium	High HALP and positive CK5/6 or Low HALP and negative CK5/6	
	High	Low HALP (HALP < = 28.447)	Positive CK5/6

*CK5/6* Cytokeratin 5/6, *EGFR* epidermal growth factor receptor, *HALP* hemoglobin, albumin, lymphocyte, and platelet, *IHC* immunohistochemistry

## Discussion

Breast cancer is a common malignant tumor with relatively high 5-year survival rate and favorable prognosis [15]. However, IBC exhibits more aggressive biological behavior and significantly poorer prognosis. In recent years, IBC has been further subclassified, and breast cancer treatment has entered the era of precision-targeted therapy. Prognostic prediction is crucial for implementing proactive measures in high-risk populations. However, single prognostic factors provide limited data. Therefore, more comprehensive and precise prognostic prediction models have become a focal point of research.

Postoperative recurrence or metastasis of IBC results from multiple factors, including tumor aggressiveness, inflammatory indicators, nutritional status, and immune response [16]. Preoperative peripheral blood parameters can reflect tumor burden, while IHC biomarkers indicate tumor aggressiveness. These parameters not only reflect the development and progression of the tumor, but also indicate the host’s capacity to respond to antitumor agents, ultimately influencing patients’ clinical survival outcomes. Thus, we hypothesized that combined models may yield a superior prognostic value.

Tumor progression is linked to an increased risk of malnutrition, highlighting the connection between tumor burden, inflammatory status, decreased caloric intake, and nutritional malabsorption [17]. The HALP integrates albumin, hemoglobin, lymphocyte, and platelet counts. This composite index reflects a patient’s nutritional status, which correlates with tumor burden. Notably, the lymphocyte and platelet components of this index provide valuable insights into host immune competence. Consequently, the HALP has emerged as a comprehensive tool for assessing patients’ baseline pathophysiological status. In recent years, the HALP has emerged as a novel prognostic indicator with predictive value for cancer outcomes in various malignancies, including colorectal, bladder, and lung cancers [18–20]. Pioneering work in 2015 first elucidated the differential prognostic correlations: hemoglobin, albumin, and lymphocyte levels exhibited positive associations with oncological outcomes, whereas platelet counts demonstrated an inverse relationship [21]. Anemia, a prevalent comorbidity often exacerbated by cancer-related inflammatory cascades, is primarily assessed through hemoglobin level measurement. Serum albumin levels are conventionally used to evaluate both nutritional and inflammatory states. Decreased lymphocyte counts coupled with elevated platelet levels may indicate compromised immunity and an increased risk of infection while chronic inflammation has been linked to various diseases, including cancer [22]. Therefore, the combined assessment of these four biochemical parameters represents a rational and feasible approach for prognostic prediction.

EGFR is a member of the epidermal growth factor receptor family and plays a key role in regulating cellular processes, such as cell proliferation and differentiation. Overexpression of EGFR can contribute to tumor-related biological behaviors, including cell proliferation, differentiation, metastasis, and angiogenesis [23, 24]. CK5/6 is a basal and luminal marker of breast cancer with pathological and prognostic significance in breast cancer [25]. EGFR and CK5/6 are crucial molecular markers for identifying basal-like breast cancer (BLBC) [26]. They exhibit co-expression patterns, and IHC expression of EGFR and CK5/6 carries significant diagnostic value for identifying BLBC and contributes importantly to the molecular subtyping of triple-negative breast cancer (TNBC) [27]. IBC, BLBC, and TNBC demonstrate higher histological malignancy and are typically associated with more rapid progression and poorer prognosis. To date, few studies have integrated preoperative peripheral blood parameters with immunohistochemical biomarkers to predict postoperative recurrence or metastasis in patients with IBC. Our study initially identified EGFR as a significant prognostic factor in premenopausal breast cancer, consistent with previous reports. However, after adjusting for molecular subtype, the effect of EGFR was no longer significant. This suggests that EGFR overexpression is closely linked to the BLBC/TNBC phenotype, and its prognostic information is largely captured by routine molecular subtyping. Nevertheless, given the limited sample size, the possibility of a modest independent effect cannot be completely excluded. Larger studies are needed to clarify whether EGFR adds independent prognostic value together with TNBC status. Notably, in our premenopausal cohort, the predictive performance of TNM stage (AUC = 0.643) was lower than that of the single EGFR biomarker (AUC = 0.701). Although this finding may appear counterintuitive at first glance, it can be reasonably explained by the following considerations. First, early-stage patients (I–II) comprised 84.6% of our cohort, limiting the discriminative ability of anatomic staging. In contrast, EGFR positivity is enriched in aggressive molecular subtypes, which confer poor prognosis even at early stages. Thus, EGFR provides prognostic information beyond TNM stage. Second, EGFR overexpression directly reflects tumor biological aggressiveness, whereas TNM stage primarily describes anatomical extent. For patients with the same stage, EGFR status can further refine risk stratification. Similar observations have been reported in other solid tumors, where molecular biomarkers outperform traditional staging in early-stage or molecularly defined subgroups [28].

BLBC is the most aggressive breast cancer subtype with the worst prognosis [29]. It exhibits a significantly higher incidence in premenopausal women compared to postmenopausal women [30]. The average onset age of BLBC is 50.7 years, markedly younger than other breast cancer subtypes. Compared with bone metastasis, BLBC has a greater propensity for viseral metastasis [31]. In this study, EGFR and CK5/6 positivity in premenopausal women with IBC was associated with postoperative recurrence and metastasis, which may be related to the earlier onset age of BLBC. Although our combined model showed improved prognostic performance, the biological interplay between the systemic HALP and tumor-intrinsic basal-like markers (EGFR, CK5/6) remains incompletely understood. A plausible link is chronic inflammation: low HALP often indicates a pro-inflammatory state, which can promote tumor progression through cytokine-mediated signaling pathways (IL-6, TNF-α) that may cross-talk with EGFR activation [32]. In BLBC, EGFR is frequently overexpressed and can be further upregulated by inflammatory stimuli, creating a vicious cycle of tumor aggressiveness and immune suppression. However, this hypothesis requires experimental validation. Notably, in our study, HALP was a significant prognostic factor only in the premenopausal cohort, not in postmenopausal patients. This discrepancy may be explained not only by the higher prevalence of BLBC in younger women, but also by age-related differences in systemic inflammation and nutritional reserve. Premenopausal women generally have more robust immune and nutritional status at baseline, making the deviation reflected by low HALP more clinically meaningful. In contrast, postmenopausal patients often have age-related chronic low-grade inflammation and nutritional decline, which could obscure the prognostic signal of HALP. Additionally, the distinct hormonal milieu may modulate the interaction between inflammatory markers and tumor biology [33]. These hypotheses warrant further investigation in larger, multi-omics cohorts.

The incremental value of our multi-parameter combined model is threefold. First, HALP demonstrated significant prognostic value specifically in premenopausal patients (OR = 0.962, *P* = 0.035), but not in postmenopausal patients (OR = 1.013, *P* = 0.102), highlighting its population-specific utility. Second, in terms of predictive performance, the HALP-EGFR combined model (AUC = 0.764) substantially outperformed HALP alone (AUC = 0.643), EGFR alone (AUC = 0.701), and TNM stage alone (AUC = 0.643), demonstrating a quantifiable incremental prognostic gain by integrating systemic inflammatory-nutritional indices with tumor biology. Third, the comprehensive model further incorporating TNM stage and CK5/6 achieved an AUC of 0.844, with bootstrap internal validation confirming its stability, suggesting promising clinical applicability for risk stratification in premenopausal breast cancer patients.

However, several limitations should be acknowledged. First, the exclusion of patients receiving neoadjuvant therapy limits the applicability of our prognostic model to the upfront surgery population. Second, the relatively high attrition rate primarily resulted from patients being lost to follow-up after transferring to local hospitals for adjuvant therapy—a common challenge in single-center studies in large geographic regions. This may have introduced selection bias toward patients with more complete follow-up data. Third, the small proportion of stage III–IV disease (15.4%) reflects local clinical practices and may not represent the full spectrum of premenopausal breast cancer. Therefore, while our model offers a promising tool for risk stratification in premenopausal breast cancer patients undergoing upfront surgery, external validation in larger, prospective, multicenter cohorts with broader inclusion criteria is essential before widespread clinical implementation. Until such validation is available, our findings should be interpreted as hypothesis-generating rather than definitive. Fourth, to assess the stability of the HALP cut-off (28.447), we performed bootstrap internal validation with 500 resamples within the premenopausal subgroup (*n* = 150). The bootstrapped optimal cut-offs showed a median of 48.54 (95% CI: 15.90–54.77, SD: 16.53), indicating that the optimal threshold varies to some extent across resamples — a phenomenon commonly observed in relatively small subgroup analyses (150 patients, 22 events). Despite this limitation, the HALP-based model using this cut-off demonstrated significant prognostic discrimination in Kaplan-Meier analysis (*P* < 0.0001), and HALP as a continuous variable remained an independent prognostic factor in multivariate Cox regression. More importantly, the primary conclusion of our study is grounded in the multi-parameter combined model rather than a single HALP threshold—the combined model (AUC = 0.844) exhibited stable and excellent predictive performance in internal validation, providing a more reliable reference for clinical decision-making. Moreover, we defined 10-year PFS as a benchmark for considering breast cancer cured; therefore, extending follow-up time and dynamically monitoring changes over time may be needed to predict long-term value.

## Conclusions

In conclusion, the novel prognostic model based on the HALP, particularly when combined with EGFR and further integrated with TNM stage and CK5/6, demonstrates high predictive value in premenopausal IBC patients, providing new insights for individualized treatment strategies in this specific population.

## Supplementary Information

Supplementary Material 1: Table S1 Binary logistic regression analysis for PFS in 310 patients with IBC. CI, confidence interval; CK5/6, Cytokeratin 5/6; EGFR, epidermal growth factor receptor; HALP, hemoglobin, albumin, lymphocyte, and platelet; IBC, invasive breast cancer; MLR, monocyte-to-lymphocyte ratio; NLR, neutrophil-to-lymphocyte ratio; OR, odds ratio; PFS, progression-free survival; PNI, prognostic nutritional index; SII, systemic immune-inflammation index. Table S2 Univariate and multivariate Cox regression analyses for PFS in premenopausal breast cancer patients (n=150). CI, confidence interval; CK5/6, Cytokeratin 5/6; EGFR, epidermal growth factor receptor; HALP, hemoglobin, albumin, lymphocyte, and platelet; HR, hazard ratio; PFS, progression-free survival. Figure S1 Kaplan-Meier curves for cancer survival in patients with IBC in the premenopausal cohort. (a) Kaplan–Meier plots of the combined model illustrating that the patients in the non-low-risk group showed poorer PFS than those in the low-risk group in HALP-EGFR model. (b) Kaplan–Meier plots of the combined model illustrating that the patients in the non-low-risk group showed poorer PFS than those in the low-risk group in EGFR-CK5/6 model. (c) Kaplan–Meier plots of the combined model illustrating that the patients in the non-low-risk group showed poorer PFS than those in the low-risk group in HALP-CK5/6 model. CK5/6, Cytokeratin 5/6; EGFR, epidermal growth factor receptor; HALP, hemoglobin, albumin, lymphocyte, and platelet; IBC, invasive breast cancer; PFS, progression-free survival. Figure S2 ROC curves and AUC of TNM-HALP-EGFR-CK5/6 model for predicting PFS. AUC, area under the curve; CK5/6, Cytokeratin 5/6; EGFR, epidermal growth factor receptor; HALP, hemoglobin, albumin, lymphocyte, and platelet; PFS: progression-free-survival; ROC, receiver operating characteristic.

## Data Availability

The datasets used and/or analysed during the current study are available from the corresponding author on reasonable request.

## Abbreviations

AUC: Area under the receiver operating characteristic curve
BLBC: Basal-like breast cancer
BMI: Body mass index
CK5/6: Cytokeratin 5/6
EGFR: Epidermal growth factor receptor
HALP: Hemoglobin-albumin-lymphocyte-platelet
IBC: Invasive breast cancer
IHC: Immunohistochemistry
MLR: Monocyte-to-lymphocyte ratio
NLR: Neutrophil-to-lymphocyte ratio
PFS: Progression-free survival
PLR: Platelet-to-lymphocyte ratio
PNI: Prognostic nutritional index
ROC: Receiver operating characteristic
SD: Standard deviation
SII: Systemic immune-inflammation index
TNBC: triple-negative breast cancer
