# Supplementary material for: A novel Hemoglobin-Albumin-Lymphocyte-Platelet (HALP)-based prognostic model to predict the progression of premenopausal breast cancer: a retrospective cohort study
Source: BMC Cancer. 2026 Jul 29;26:1294. doi: 10.1186/s12885-026-16642-7 (PMC13700329; doi:10.1186/s12885-026-16642-7)
Supplement: Supplementary file 1 [file 12885_2026_16642_MOESM1_ESM.docx]

**Supplementary Materials**

**Table S1** Binary logistic regression analysis for PFS in 310 patients with IBC

| **Cohort** | **Indicators** | | **OR (95% CI)** | | ***P* value** | |
| --- | --- | --- | --- | --- | --- | --- |
| **Total** | EGFR | | 3.516 (1.712–7.220) | | 0.001 | |
|  | CK5/6 | | 2.64 (1.231–5.661) | | 0.013 | |
|  | HALP | | 1.005 (0.994–1.016) | | 0.393 | |
|  | PNI | | 0.962 (0.889–1.040) | | 0.328 | |
|  | MLR | | 1.172 (0.110-12.539) | | 0.895 | |
|  | PLR | | 1.001 (0.995–1.007) | | 0.732 | |
|  | NLR | | 0.963 (0.845–1.098) | | 0.575 | |
|  | SII | | 1 (0.999-1.000) | | 0.719 | |
| **Cohort** | | **Indicators** | | **OR (95% CI)** | | ***P* value** |
| **BMI < 24** | | EGFR | | 3.378 (0.937–12.179) | | 0.063 |
|  |  | CK5/6 | | 2.701 (0.728–10.029) | | 0.138 |
|  |  | HALP | | 0.933 (0.884–0.985) | | 0.012 |
|  |  | PNI | | 0.91 (0.787–1.053) | | 0.205 |
|  |  | MLR | | 0.279 (0.002–31.441) | | 0.596 |
|  |  | PLR | | 1.006 (0.999–1.013) | | 0.095 |
|  |  | NLR | | 1.076 (0.873–1.327) | | 0.491 |
|  |  | SII | | 1.001(1.000-1.001) | | 0.206 |
| **BMI > = 24** | | EGFR | | 3.556 (1.480–8.542) | | 0.005 |
|  |  | CK5/6 | | 2.822 (1.084–7.349) | | 0.034 |
|  |  | HALP | | 1.009 (1.996–1.021) | | 0.163 |
|  |  | PNI | | 0.977 (0.889–1.075) | | 0.639 |
|  |  | MLR | | 2.752 (0.186–40.709) | | 0.462 |
|  |  | PLR | | 0.996 (0.986–1.005) | | 0.345 |
|  |  | NLR | | 0.917 (0.759–1.108) | | 0.369 |
|  |  | SII | | 0.999 (0.998-1.000) | | 0.245 |

| **Cohort** | **Indicators** | **OR (95% CI)** | ***P* value** |
| --- | --- | --- | --- |
| **Age < 45** | EGFR | 4.25 (1.125–16.060) | 0.033 |
|  | CK5/6 | 3.452 (1.920-12.957) | 0.066 |
|  | HALP | 0.896 (0.832–0.965) | 0.004 |
|  | PNI | 0.893 (0.775–1.030) | 0.893 |
|  | MLR | 0.432 (0.008–22.086) | 0.676 |
|  | PLR | 1.021 (1.005–1.036) | 0.008 |
|  | NLR | 1.034 (0.899–1.190) | 0.64 |
|  | SII | 1 (1.000-1.001) | 0.256 |
| **Age ≥ 45** | EGFR | 3.312 (1.405–7.808) | 0.006 |
|  | CK5/6 | 2.276 (0.877–5.910) | 0.091 |
|  | HALP | 1.011 (0.998–1.024) | 1.11 |
|  | PNI | 0.999 (0.906–1.101) | 0.982 |
|  | MLR | 2.225 (0.099–50.151) | 0.615 |
|  | PLR | 0.996 (0.987–1.004) | 0.314 |
|  | NLR | 0.856 (0.671–1.091) | 0.209 |
|  | SII | 0.999 (0.998-1.000) | 0.181 |

| **Cohort** | **Indicators** | **OR (95% CI)** | ***P* value** |
| --- | --- | --- | --- |
| **N-** | EGFR | 1.432 (0.370–5.545) | 0.604 |
|  | CK5/6 | 1.397 (0.275–7.094) | 0.687 |
|  | HALP | 0.969 (0.922–1.018) | 0.21 |
|  | PNI | 1.014 (0.871–1.181) | 0.858 |
|  | MLR | 0.076 (0.000-49.211) | 0.436 |
|  | PLR | 1.004(0.996–1.012) | 0.383 |
|  | NLR | 0.865 (0.590–1.269) | 0.459 |
|  | SII | 0.999 (0.998–1.001) | 0.514 |
| **N+** | EGFR | 5.203 (2.097–12.910) | < 0.0001 |
|  | CK5/6 | 3.667 (1.426–9.431) | 0.007 |
|  | HALP | 1.006 (0.994–1.017) | 0.332 |
|  | PNI | 0.935 (0.850–1.029) | 0.17 |
|  | MLR | 1.533 (0.102–23.104) | 0.758 |
|  | PLR | 0.999 (0.991–1.008) | 0.904 |
|  | NLR | 0.972 (0.847–1.115) | 0.681 |
|  | SII | 1 (0.999–1.001) | 0.889 |

| **Cohort** | **Indicators** | **OR (95% CI)** | ***P* value** |
| --- | --- | --- | --- |
| **Luminal** | EGFR | 2.222 (0.888–5.563) | 0.088 |
|  | CK5/6 | 1.532 (0.416–5.635) | 0.521 |
|  | HALP | 1.009 (0.997–1.020) | 0.142 |
|  | PNI | 0.976 (0.883–1.080) | 0.638 |
|  | MLR | 0.351 (0.009–13.773) | 0.576 |
|  | PLR | 1.000 (0.993–1.008) | 0.963 |
|  | NLR | 0.859 (0.663–1.113) | 0.251 |
|  | SII | 0.999 (0.998-1.000) | 0.275 |
| **HER2+** | EGFR | 6 (0.689–52.266) | 0.105 |
|  | CK5/6 | 1.833 (0.484–6.948) | 0.373 |
|  | HALP | 0.992 (0.949–1.038) | 0.735 |
|  | PNI | 1 (0.853–1.173) | 1.000 |
|  | MLR | 0.854 (0.012–58.680) | 0.942 |
|  | PLR | 1.004 (0.989–1.019) | 0.618 |
|  | NLR | 0.034 (0.821–1.301) | 0.777 |
|  | SII | 1.000 (0.999–1.001) | 0.781 |
| **TNBC** | EGFR | 358994401 (0) | 0.999 |
|  | CK5/6 | 5.333 (0.481–59.144) | 0.173 |
|  | HALP | 0.893 (0.793–1.005) | 0.061 |
|  | PNI | 0.769 (0.580–1.020) | 0.069 |
|  | MLR | 155.446 (0.128-189277.003) | 0.164 |
|  | PLR | 1.008 (0.986–1.031) | 0.481 |
|  | NLR | 1.229 (0.810–1.865) | 0.333 |
|  | SII | 1.002 (1.000-1.004) | 0.106 |

| **Cohort** | **Indicators** | **OR (95% CI)** | ***P* value** |
| --- | --- | --- | --- |
| **Ki-67 < 14** | EGFR | 4.556 (0.388–53.514) | 0.228 |
|  | CK5/6 | 3.187 (0.258–39.360) | 0.366 |
|  | HALP | 1.009 (0.935–1.089) | 0.816 |
|  | PNI | 1.066 (0.820–1.387) | 0.632 |
|  | MLR | 0.515 (0.000-2609.856) | 0.879 |
|  | PLR | 1.001 (0.982–1.019) | 0.946 |
|  | NLR | 0.559 (0.123–2.552) | 0.453 |
|  | SII | 0.998 (0.993–1.004) | 0.557 |
| **Ki-67 ≥ 14** | EGFR | 3.329 (1.561–7.097) | 0.002 |
|  | CK5/6 | 2.532 (1.130–5.672) | 0.024 |
|  | HALP | 1.005 (0.994–1.016) | 0.414 |
|  | PNI | 0.947 (0.873–1.028) | 0.196 |
|  | MLR | 1.283 (0.102–16.201) | 0.847 |
|  | PLR | 1.001 (0.994–1.007) | 0.82 |
|  | NLR | 0.967 (0.846–1.106) | 0.628 |
|  | SII | 1 (0.999–1.001) | 0.776 |

| **Cohort** | **Indicators** | **OR (95% CI)** | ***P* value** |
| --- | --- | --- | --- |
| **Premenopausal** | EGFR | 5.924 (2.225–15.772) | < 0.0001 |
|  | CK5/6 | 3.738 (1.411–9.908) | 0.008 |
|  | HALP | 0.962 (0.928–0.997) | 0.035 |
|  | PNI | 0.953 (0.863–1.053) | 0.348 |
|  | MLR | 0.977 (0.060-15.838) | 0.987 |
|  | PLR | 1.010 (1.000-1.020) | 0.043 |
|  | NLR | 1.002 (0.875–1.147) | 0.981 |
|  | SII | 1.000 (0.999–1.001) | 0.716 |
| **Postmenopausal** | EGFR | 2.125 (0.718–6.286) | 0.173 |
|  | CK5/6 | 1.398 (0.365–5.360) | 0.625 |
|  | HALP | 1.013 (0.997–1.029) | 0.102 |
|  | PNI | 0.967 (0.853–1.097) | 0.606 |
|  | MLR | 1.354 (0.017-109.272) | 0.893 |
|  | PLR | 0.991 (0.978–1.004) | 0.161 |
|  | NLR | 0.835 (0.601–1.159) | 0.281 |
|  | SII | 0.999 (0.998–1.001) | 0.277 |

| **Cohort** | **Indicators** | **OR (95% CI)** | ***P* value** |
| --- | --- | --- | --- |
| **HR+** | EGFR | 2.222 (0.888–5.563) | 0.088 |
|  | CK5/6 | 1.532 (0.416–5.635) | 0.521 |
|  | HALP | 1.009 (0.997–1.020) | 0.142 |
|  | PNI | 0.976 (0.883–1.080) | 0.638 |
|  | MLR | 0.351 (0.009–13.773) | 0.576 |
|  | PLR | 1.000 (0.993–1.008) | 0.963 |
|  | NLR | 0.859 (0.663–1.113) | 0.251 |
|  | SII | 0.999 (0.998-1.000) | 0.275 |
| **HR-** | EGFR | 7.125 (0.875–57.987) | 0.066 |
|  | CK5/6 | 2.327 (0.757–7.147) | 0.14 |
|  | HALP | 0.97 (0.933–1.009) | 0.126 |
|  | PNI | 0.922 (0.809–1.050) | 0.219 |
|  | MLR | 3.444 (0.123–96.086) | 0.467 |
|  | PLR | 1.005 (0.993–1.018) | 0.392 |
|  | NLR | 1.069 (0.876–1.304) | 0.511 |
|  | SII | 1.000 (1.000-1.001) | 0.332 |

CI, confidence interval; CK5/6, Cytokeratin 5/6; EGFR, epidermal growth factor receptor; HALP, hemoglobin, albumin, lymphocyte, and platelet; IBC, invasive breast cancer; MLR, monocyte-to-lymphocyte ratio; NLR, neutrophil-to-lymphocyte ratio; OR, odds ratio; PFS, progression-free survival; PNI, prognostic nutritional index; SII, systemic immune-inflammation index.

**Table S2** Univariate and multivariate Cox regression analyses for PFS in premenopausal breast cancer patients (n = 150)

| **Group** | **HR (95% CI, non-adjusted)** | ***P* value** | **HR (95% CI, adjusted)** | ***P* value** |
| --- | --- | --- | --- | --- |
| TNM stage | 2.33 (1.26–4.28) | 0.007 | 3.8 (1.81–7.99) | 0.0005 |
| Ki-67 (< 14 VS ≥ 14) | 3.3 (0.42–25.71) | 0.255 | 1.84 (0.22–15.26) | 0.573 |
| HALP (low VS high) | 0.25 (0.1–0.64) | 0.004 | 0.26 (0.1–0.68) | 0.006 |
| EGFR (+/-) | 4.99 (1.72–14.51) | 0.003 | 2.83 (0.9–8.96) | 0.076 |
| CK5/6 (+/-) | 1.98 (0.71–5.54) | 0.194 | 1.18 (0.39–3.54) | 0.764 |
| BMI (< 24 VS ≥ 24) | 0.71 (0.27–1.82) | 0.473 | 0.36 (0.13–1.03) | 0.06 |
| Age (< 45 VS ≥ 45) | 2.32 (0.85–6.31) | 0.099 | 3.3 (1.12–9.72) | 0.030 |

CI, confidence interval; CK5/6, Cytokeratin 5/6; EGFR, epidermal growth factor receptor; HALP, hemoglobin, albumin, lymphocyte, and platelet; HR, hazard ratio; PFS, progression-free survival.

**Figure S1** Kaplan-Meier curves for cancer survival in patients with IBC in the premenopausal cohort. (a) Kaplan–Meier plots of the combined model illustrating that the patients in the non-low-risk group showed poorer PFS than those in the low-risk group in HALP-EGFR model. (b) Kaplan–Meier plots of the combined model illustrating that the patients in the non-low-risk group showed poorer PFS than those in the low-risk group in EGFR-CK5/6 model. (c) Kaplan–Meier plots of the combined model illustrating that the patients in the non-low-risk group showed poorer PFS than those in the low-risk group in HALP-CK5/6 model. CK5/6, Cytokeratin 5/6; EGFR, epidermal growth factor receptor; HALP, hemoglobin, albumin, lymphocyte, and platelet; IBC, invasive breast cancer; PFS, progression-free survival.

**Figure S2** ROC curves and AUC of TNM-HALP-EGFR-CK5/6 model for predicting PFS. AUC, area under the curve; CK5/6, Cytokeratin 5/6; EGFR, epidermal growth factor receptor; HALP, hemoglobin, albumin, lymphocyte, and platelet; PFS: progression-free-survival; ROC, receiver operating characteristic.
